# Metformin Toxicity Unmasked by Obstructive Uropathy: A Case of Metformin Associated Lactic Acidosis and Euglycemic Diabetic Ketoacidosis

**DOI:** 10.15190/d.2026.6

**Published:** 2026-04-01

**Authors:** Rubba Shoukat Khan, Ashina Patla, Meher Ayyazuddin, George Michaeil, Rehan Shah, Sujanthy Rajaram

**Affiliations:** ^1^Department of Medicine, Bayonne University Hospital, Hudson Regional Health, Bayonne, NJ, USA

**Keywords:** Metformin-associated lactic acidosis, Euglycemic DKA, Refractory lactic acidosis.

## Abstract

Metformin-associated lactic acidosis (MALA) is a rare but life-threatening complication of metformin therapy that most commonly occurs in the setting of acute kidney injury (AKI) and impaired drug clearance. Given the widespread use of metformin for type 2 diabetes mellitus, recognition of precipitating factors for MALA remains critically important. We report a case of severe euglycemic diabetic ketoacidosis (DKA) with refractory lactic acidosis in a patient with previously unrecognized obstructive uropathy due to recurrent nephrolithiasis causing bilateral hydronephrosis and severe AKI while on metformin therapy. The patient developed profound metabolic derangements that were unresponsive to conventional medical therapy, with resolution only after emergent hemodialysis. This case highlights the importance of early recognition of AKI from obstructive uropathy as a reversible precipitant of metformin accumulation and emphasizes the role of prompt renal replacement therapy in patients with severe MALA and refractory metabolic acidosis.

## 1. Introduction

Metformin is a widely prescribed first-line oral hypoglycemic agent for the management of type 2 diabetes mellitus due to its efficacy, low cost, and cardiovascular benefits^1^. Metformin-associated lactic acidosis is rare with an estimated incidence of 6.3 cases per 100,000 patient-years^2,3^. The drug is primarily eliminated unchanged by the kidneys via glomerular filtration and active tubular secretion. Thus, risk of MALA is significantly increased in the presence of conditions that impair renal clearance of metformin^4,5^.

AKI in the setting of obstructive uropathy is most often attributable to stone-induced obstruction of a functioning kidney, accounting for approximately 10% of AKI cases. This may predispose to toxic accumulation of the drug and subsequent lactic acidosis^6^. Although MALA is rare, close monitoring of renal function is essential to enable timely recognition and discontinuation of the drug.

We present the case of a 66-year-old male with type 2 diabetes mellitus on metformin who developed MALA with euglycemic DKA in the setting of severe AKI secondary to obstructive uropathy, emphasizing the need for vigilance in similar high-risk clinical situations.

## 2. Case presentation

**Figure 1:**
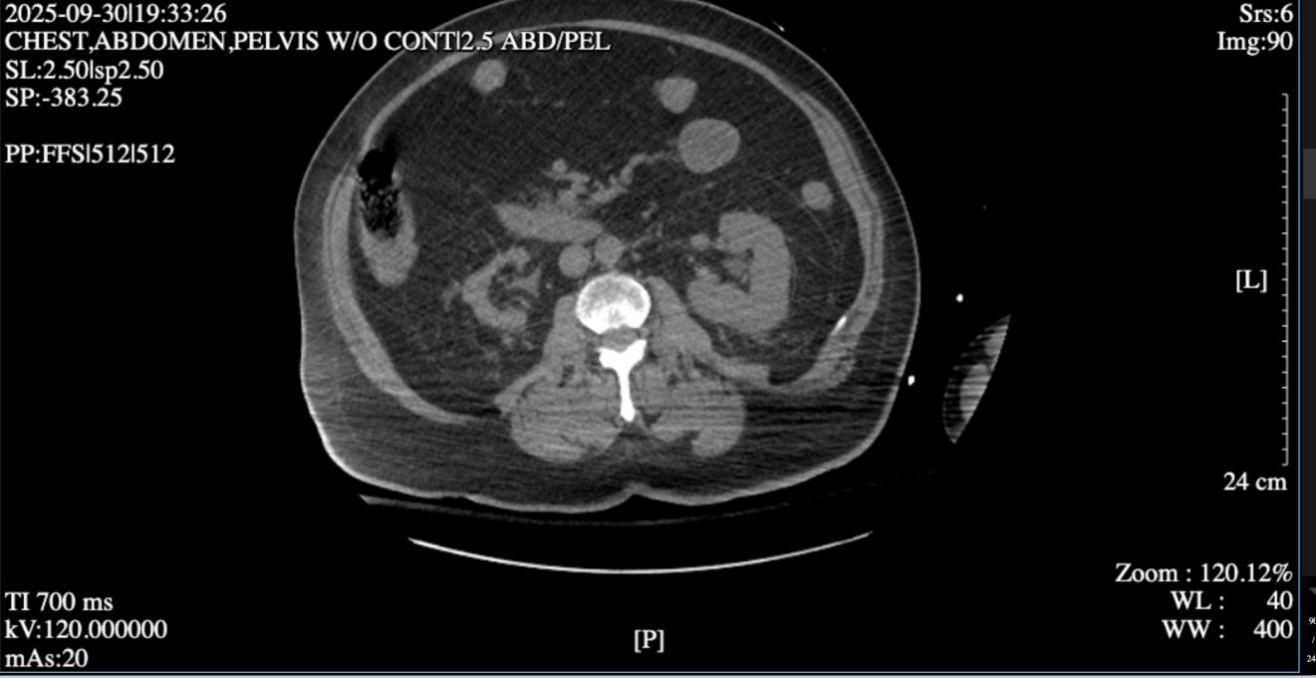
CT scan of the abdomen and pelvis: obstructing 3mm calculus in the proximal left ureter with mild left hydronephrosis; colonic diverticulosis without evidence of diverticulitis

A 66-year-old male with a past medical history of well-controlled type 2 diabetes mellitus (DM2), coronary artery disease (CAD) with prior stent placement, hypertension, hyperlipidemia, and recurrent nephrolithiasis presented with a two-day history of worsening shortness of breath and generalized weakness. He reported anuria for four days, poor appetite, and left flank pain for one week. His diabetes had been managed with metformin monotherapy. He was not taking any SGLT2 inhibitors such as dapagliflozin or empagliflozin for his DM2; this is noteworthy as fasting and withholding SGLT2 inhibitors can also precipitate euglycemic DKA, which was excluded in this case. In the emergency department, he was tachycardic with Kussmaul respirations and required positive airway pressure support. Laboratory evaluation revealed a high anion gap metabolic acidosis (anion gap 42) with lactate 5.7 mmol/L, beta-hydroxybutyrate 11.8 mmol/L, pH 7.01, pCO₂ 12 mmHg, bicarbonate <5 mmol/L, and serum glucose 196 mg/dL, consistent with euglycemic DKA. He also had AKI with creatinine 14.6 mg/dL (baseline 1.3 mg/dL), eGFR 3 mL/min/1.73 m², BUN 93 mg/dL, and potassium 7.3 mmol/L. Hyperkalemia was promptly treated, and aggressive fluid resuscitation for DKA was initiated. A CT scan of the abdomen and pelvis showed an obstructing 3 mm calculus in the proximal left ureter with mild left hydronephrosis and colonic diverticulosis (**Figure 1**). Urology was consulted, and conservative management with hydration, pain control, and medical expulsive therapy was initiated. No invasive intervention was required as the patient remained hemodynamically stable. By hospital day one, BUN decreased to 55 mg/dL and creatinine to 12.3 mg/dL, with urine output of 2.4 liters, indicating improving renal function. On repeat ultrasound two days later, there was no hydronephrosis, suggesting that the stone had passed spontaneously.

Although DKA resolved within 48 hrs with a negative repeat beta-hydroxybutyrate level, lactic acidosis persisted (lactate 5.7 mmol/L, pH 7.01, AG 23) despite adequate resuscitation and resolution of ketosis. Septic workup including cultures and procalcitonin was negative. The patient was consistently afebrile and given his ongoing metformin therapy and renal dysfunction, MALA was suspected. The patient underwent euvolemic hemodialysis for the refractory acidosis, hyperkalemia, severe AKI with GFR 3 mL/min/1.73 m², after which lactate normalized to 0.9 mmol/L, creatinine improved to 6.2 mg/dL, and hydronephrosis resolved on follow-up imaging.

Metformin was discontinued permanently at discharge due to MALA risk. He was transitioned to repaglinide 0.5 mg before meals for glycemic management. Lisinopril was previously held during hospitalization due to AKI, was not restarted at discharge pending renal recovery and was to be reassessed by his primary care physician.

At discharge, the patient was clinically stable with improving renal function. He was provided with outpatient referrals to endocrinology for diabetes optimization and possible initiation of alternative glucose-lowering therapy, nephrology for renal function monitoring, and urology for follow-up regarding nephrolithiasis prevention.

## 3. Discussion

This case highlights the interplay between impaired renal clearance and drug-induced toxicity. Metformin remains the cornerstone of first-line therapy for type 2 diabetes and is among the most frequently prescribed medications worldwide^1,3^. In this case, the presence of one chronically atrophic kidney due to recurrent nephrolithiasis and an acutely obstructed contralateral kidney with hydronephrosis causes ongoing obstruction, GFR decline and proximal convoluted tubule injury, leading to disruption of normal reabsorptive mechanisms and impaired toxin clearance^7^. Therapeutic and toxic ranges of metformin have been established through clinical and forensic studies, and blood concentration of metformin is highly dependent on kidney function^8^.

It is important to note that, in addition to metformin, the patient was also on valsartan, an angiotensin receptor blocker known to impair renal function by increasing serum creatinine and potassium through reduced renal perfusion. This may have further burdened the already compromised kidneys, promoting metformin accumulation^9^. To manage the hyperkalemia, the patient was initiated on a low-dose insulin drip; however, the elevated anion gap remained refractory to treatment. This strongly suggested that, despite appropriate management of euglycemic DKA, lactic acidosis was a major contributor to the persistent anion gap metabolic acidosis. As highlighted by Feras Al Moussaly et al., the coexistence of lactic acidosis with euglycemic ketosis is exceedingly rare, and a strong association has been observed when metformin levels exceed 9.9 mg/L^10,11^.

The pathogenesis of MALA includes both lactate overproduction and impaired drug clearance with inhibition of hepatic gluconeogenesis and suppression of mitochondrial oxidative phosphorylation along with inhibition of pyruvate carboxylase^12^. Early initiation of renal replacement therapy, particularly hemodialysis, is recognized as the mainstay of treatment for MALA, as it efficiently facilitates metformin clearance and correction of acidosis^13^. In our patient, there was major improvement in anion gap, acidosis and lactate clearance after the first dialysis session.

As per recommendations, a patient with declining GFR should only be on metformin therapy if the patient’s eGFR is above 30 ml/min/1.73m^3^ ^14,15^. MALA is associated with a mortality rate of up to 50%, with worse outcomes correlating with the severity of acidosis and the degree of hyperlactatemia^16^.Vigilant monitoring of kidney functioning is required and stop metformin if eGFR drops below 30ml/min/1.73m^3^. Changing the medication regimen to a more appropriate therapy to prevent prevalence of adverse events would be a suitable course of action. Hussein et al supports the timely recognition of specific settings like dehydration, gastroenteritis, renal impairment and overdose to decrease the risk of metformin accumulation and associated poor outcomes^17^.

The limitation of our report includes the fact that serum metformin was not measured before dialysis. The patient was scheduled for two sessions, and the prognostic value of therapy could not be gauged. However, a high level of metformin is needed to cause lactic acidosis so it may be understood that the metformin levels were critically high and after dialysis, the lactate proved to clear with improvement in acidosis. Serum metformin levels are not routinely measured and, in this case, possibly metformin accumulation caused lactic acidosis which was proved by clearance of acidosis after hemodialysis.

## 4. Conclusion

This case highlights the critical association between acute kidney injury and chronically compromised renal function in patients receiving metformin therapy. In individuals with diabetes who have underlying obstructive uropathy or structural renal abnormalities such as an atrophic kidney, the risk of metformin accumulation and subsequent lactic acidosis may be significantly increased. Therefore, metformin use in these patients should be closely monitored through regular assessment of renal function, particularly eGFR. A multidisciplinary approach involving primary care, nephrology, and endocrinology may help ensure appropriate surveillance and timely discontinuation of metformin when renal function declines, thereby reducing the risk of life-threatening metabolic complications.

## Acknowledgements

We would like to thank our Program for their continuous support and guidance in the preparation of this work. We also extend our gratitude to the entire critical care team for their invaluable clinical expertise and collaborative input in the management of this patient. No funding was received for this study.

## Consent

Informed consent was obtained from the patient for publication of this case report and accompanying images.

## Author Contributions

Rubba S Khan conceptualized the study and contributed to the abstract, introduction, manuscript drafting, and critical review and editing. Meher Ayyazuddin contributed to the introduction and discussion, and was involved in manuscript review, final editing, and compilation. Ashina Patla prepared the case presentation. George Michaeil was responsible for image acquisition and reference management. Dr Shah and Dr Rajaram served as senior authors, providing oversight, critical review, editing, and assistance with manuscript compilation.

## AI Statement

No AI was used in any aspect of this manuscript compilation

## Publisher’s note

All claims expressed in this article are solely those of the authors and do not necessarily represent those of their affiliated organizations, or those of the publisher, the editors and the reviewers. Any product that may be evaluated in this article, or claim that may be made by its manufacturer, is not guaranteed or endorsed by the publisher.
